# Uneven distribution of complementary sex determiner (*csd*) alleles in *Apis mellifera* population

**DOI:** 10.1038/s41598-017-02629-9

**Published:** 2017-05-24

**Authors:** Joanna Zareba, Pawel Blazej, Agnieszka Laszkiewicz, Lukasz Sniezewski, Michal Majkowski, Sylwia Janik, Malgorzata Cebrat

**Affiliations:** 1 Laboratory of Molecular and Cellular Immunology, Hirszfeld Institute of Immunology and Experimental Therapy, Polish Academy of Sciences, Weigla 12, 53-114 Wroclaw, Poland; 2 Department of Genomics, Faculty of Biotechnology, Wroclaw University, F. Joliot-Curie 14a, 50-383 Wroclaw, Poland

## Abstract

The complementary sex determiner (*csd*) gene determines the sex of the western honey bee (*Apis mellifera* L.). Bees that are heterozygous at the *csd* locus develop into females; whereas hemizygous bees develop into males. The co-occurrence of two identical *csd* alleles in a single diploid genome leads to the genetic death of the bee. Thus, the maintenance of *csd* diversity in the population is favoured. The number and distribution of *csd* alleles is particularly interesting in light of the recent decline in the honey bee population. In this study, we analysed the distribution of *csd* alleles in two Polish populations separated by about 100 km. We analysed the maternal alleles of 193 colonies and found 121 different alleles. We also analysed the distribution and frequency of the alleles, and found that they are distributed unevenly. We show that the methods that have been used so far to estimate the total worldwide number of *csd* alleles have significantly underestimated their diversity. We also show that the uneven distribution of *csd* alleles is caused by a large number of infrequent alleles, which most likely results from the fact that these alleles are generated very frequently.

## Introduction

Western honey bees, like other *Hymenoptera*, are haplodiploids, which means that the males develop from unfertilised oocytes and are haploid; whereas the females develop from fertilised oocytes and are diploid^1^. It is widely accepted that this strategy of reproduction, on the one hand, helps to remove deleterious recessive mutations from the population’s gene pool through the death of the haploid males that harbour these mutations^2, 3^. On the other hand, this is also dangerous due to a faster decline of the population under conditions that will affect its genetic diversity^4, 5^. The key factor that is responsible for the sex determination in honey bees is the complementary sex determiner (*csd*) gene^6^. Bees that are heterozygous at the *csd* locus develop into females; whereas hemizygous bees develop into males. In relatively rare cases, the sperm and the egg cell contain identical *csd* alleles, and a diploid male forms. The colony destroys all diploid males during the early stages of their development^7–9^ and therefore the emergence of diploid males is unfavourable for the colony due to the reduction in the number of worker bees and the resulting weakening of the colony. The risk of the co-occurrence of functionally identical *csd* alleles in a single genome is reduced by polyandry (in natural conditions, the queen is inseminated by several drones), as well as by behavioural traits that limit the possibility of sib-mating and, possibly, the preselection of gametes^2, 3^. Nonetheless, the key factor that is responsible for optimal reproduction is the maintenance of a high diversity of *csd* alleles in the bee population^10, 11^.

**Figure 1:**
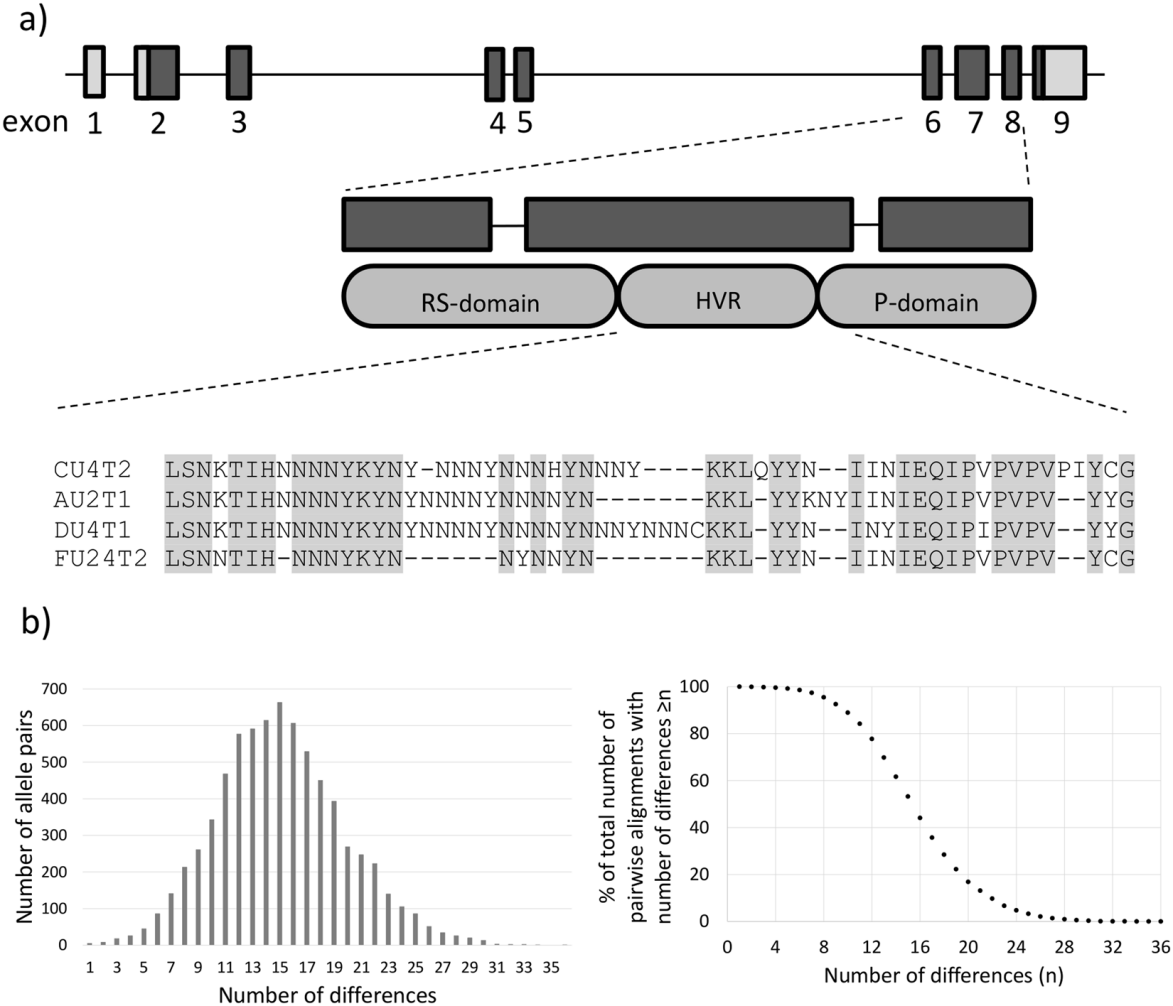
*Apis mellifera csd* locus. (**a**) Schematic representation of the *Apis mellifera csd* locus and the location of the elements that encode the potential specifying domain (PSD) containing: arginine/serine-rich domain (RS-domain), hypervariable region (HVR) and proline-rich domain (P-domain). A representative alignment of the HVR for four *csd* sequences is shown. The range of the analysed *csd* sequence is marked. (**b**) Distribution of the number of differences observed between every two *csd* sequences that were obtained during a pairwise alignment of all the 121 non-identical *csd* sequences, which were identified in Population AB (left diagram), and the fraction of the total number of pairwise alignments with the number of differences ≥n (right diagram).

The strong selection against *csd* homozygotes is advantageous for newly-formed *csd* alleles, because the probability that a new allele can create homozygotes is much lower than it is for alleles that are already present in a given population. The probability of forming homozygotes increases with the prevalence of a given allele in the population, which is why the *csd* gene evolves under balancing (negative frequency-dependent) selection^12^. The *csd* gene consists of 9 exons, of which exons 6 to 8 encode the potential specifying domain (*csd*-PSD) (Fig. 1a)^6^. This domain has been identified as a target for balancing selection^13^. In contrast to the *csd*-PSD, the gene fragment that encodes the N-terminal part of the protein accumulates far fewer nucleotide polymorphisms and has been proven to be a target for purifying selection. The *csd*-PSD also contains a hypervariable region (HVR), which consists of a variable number of A/T-rich nucleotide repeats, and encodes mainly tyrosine and asparagine residues. It is flanked by the domains that encode the arginine/serine- and proline-rich regions, both of which likely take part in protein-protein interactions. However, the molecular mechanism that underlies the functions of the *csd* gene has only been partially described. In heterozygotes, the csd protein(s), together with *Am-tra2* (a protein that contains the RNA-binding domain), direct the female-specific splicing of *fem* transcripts, which create the protein product that is responsible for the female-specific splicing of the *Am-dsx* transcript. A lack of csd protein activity (in homo- or hemi-allelic organisms, or during the downregulation of *csd* expression) results in male-specific *fem* splicing, which in turn results in a transcript that contains a stop codon in exon 3. Consequently, the translation is terminated prematurely, and the *Am-dsx* is spliced in a male-specific manner, producing a protein with a male-specific carboxy-terminal end^14–17^.

The existence of *csd* alleles that differ in terms of the sequence of HVR, but not in the sequences of the adjacent regions, suggests that the HVR is evolving at a high rate. The mechanism that is responsible for the variability of the HVR sequence is likely similar to the mechanism responsible for microsatellite variability. Single and multiple triplet changes (TAA and TAT), which often occur as sets of motifs, indicate duplications and/or unequal crossing-over. In addition, a single nucleotide polymorphism can occur, leading to amino acid substitutions. Lechner *et al*. have shown that the evolutionary processes that lead to the variations in the length of *csd* HVR are as much as 2.4-times faster than the corresponding processes in microsatellites. This leads to the conclusion that HVR represents a mutational hotspot^18^. The role of HVR variation as a factor that differentiates *csd* alleles has been omitted in research studies for a long time. Only recent studies have shown that this variation is the main, or even a sufficient, factor responsible for the generation of new *csd* specificities^18, 19^.

The aim of our study was to analyse the hypervariable regions of *csd* alleles in two distinct honey bee populations, in order to gain an insight into the distribution and frequency of these alleles. The results show that the *csd* alleles are not distributed uniformly, and that the methods that have been used in studies so far have underestimated the worldwide number of *csd* alleles, which raises new questions concerning rate of their emergence and propagation in the honey bee population.

## Results and Discussion

### Determination of the diversity of *csd* alleles in the analysed populations

We analysed maternal *csd* alleles of honey bee colonies that were kept in relatively small apiaries (6–25 colonies in each), which were located in two regions of south-western Poland. The first group (Group A) included 6 apiaries located near Wroclaw; while the second group (Group B) included 12 apiaries located near Ladek-Zdroj, about 100 km away from Group A. The apiaries in Group A were located in a relatively small area (a radius of 3.5 km), whereas the apiaries in Group B were scattered over a wider area (a radius of about 20 km). The distance between the two groups of apiaries was planned to be large enough to exclude the direct mating of bees during a single breeding season. We then collected 5 or 6 drone larvae or pupae from 99 colonies of Group A, and from 94 colonies of Group B, in order to identify the maternal *csd* alleles that were present in a given colony. Assuming random assortment of *csd* alleles in the unfertilised eggs, an analysis of six drone haplotypes would be sufficient to identify the alleles of both queens with a probability higher than 0.95. Next we amplified the genomic DNA containing fragments of the *csd* gene that encoded the HVR and part of the proline-rich domain (Fig. 1a) using the nested PCR reaction. The terminal restriction fragment length polymorphism method was applied to discriminate amplicons that represented the two alleles carried by the queen from each colony, which allowed for the sequencing of only a single representative of each allele. As a result, we obtained 184 *csd* sequences from the Group A colonies and 188 *csd* sequences from Group B. Next, we translated the obtained sequences *in silico* and trimmed them to contain only the fragment encoded by exon 7, which represented the hypervariable region and part of the proline-rich domain of the *csd* protein (Fig. 1a). The amino acid sequences were then aligned, in order to determine the number of non-identical sequences. The set of non-identical nucleotide sequences identified in this study has been deposited in GenBank (www.ncbi.nlm.nih.gov/genbank) under the accession numbers KY008252–KY008391.

### Analysis of differences in the identified *csd* sequences

An analysis of the data obtained from both groups (A and B) identified 121 non-identical csd protein sequences. Next, we performed a pairwise alignment of this set of sequences, and obtained 7260 alignments of the potential combinations of *csd* alleles. We then calculated the total number of differences (represented by the difference in the HVR lengths and the number of amino acid substitutions) between the sequences in each alignment. The differences ranged from 1 to 36 positions, with 95% of the sequence combinations differing in the range of 7 to 26 positions (Fig. 1b, left).

It is reasonable to assume that the pairs of *csd* alleles that exhibited small pairwise differences in their sequences would not have established a functional pair of alleles, i.e. they would not have determined the female development of the embryo. Lechner *et al*.^18^ attempted to determine the criteria for functional heterozygosity by analysing the differences between 77 pairs of *csd* alleles that were found in natural female genotypes. They proposed that the minimal requirements for the functional differences of *csd* alleles be given by the following criteria: d_HVR_ ≥ 6, d_PSD_ ≥ 1 and 3d_PSD_ + 2d_e8_ ≥ 9, where: d_HVR_ is the difference in the length of the HVR region; d_PSD_ is the number of amino acid mismatches in the PSD region; and d_e8_ is the number of amino acid mismatches in the part of the protein that is encoded by exon 8.

Although these results may help to determine the conditions needed to establish the functional heterozygosity of *csd* alleles, it seems that these criteria should not be considered as definitive. We think that further insights could be gained by gathering information on a far greater number of *csd* alleles and the allele pairs that are present in a given population, and determining which of the *csd* alleles that exist within this population cannot be found as a given pair in females present in population. Additionally, a later paper by Beye *et al*. shows that a pair of *csd* alelles that differs by only a 5 amino acid indel in the HVR region is able, albeit not with full penetrance, to determine the female development of the embryo^19^. However, it is important to note that only one such pair of nearly identical alleles that were unable to determine the full penetrance of femaleness has been described so far, and that research has never shown that the allele pairs that do not meet the criteria described by Lechner *et al*. are unable to establish functional heterozygosity, i.e. that a pair of non-identical *csd* alleles is only able to determine the development of diploid males.

Therefore, following the findings of Beye *et al*., and in the absence of additional data, we provisionally assumed that a difference of 5 amino acids in the HVR was the minimal difference needed for functional heterozygosity. We found that 99.2% of the pairwise combinations of *csd* alleles present in our population met this criterion (Fig. 1b, right). In fact, more than 95% of the allele pairs had ≥8 differences in the HVR, which suggests that virtually all of the combinations of *csd* alleles that were identified in our population are functional. Therefore, we decided to treat all of the 121 identified non-identical *csd* sequences as different alleles.

Regardless of the adopted criteria of functional heterozygosity, we would also like to emphasise that in our opinion, it is more appropriate to estimate the ‘functional potential’ of the total combinations of *csd* alleles that are present in a given population, rather than to estimate the number of ‘functionally distinct’ alleles^18^. Our approach has the advantage of taking into account the possibility that any two alleles that cannot form a functional pair may have the different potential to create a functional pair with a given third allele. Thus, it is clear that the differences between the *csd* alleles creates a continuum; and also, it is impossible to separate these alleles into distinct groups with a given specificity.

### Analysis of the geographical distribution of *csd* alleles

We identified 85 and 74 different *csd* alleles in Groups A and B, respectively. We then calculated the expected minimal number of different alleles (confidence level ≥0.95) (see the Materials and Methods section for a description of the estimation method) under the assumption that the *csd* alleles were uniformly distributed, at least locally. The expected number of different *csd* alleles was estimated at 111 and 78 for Populations A and B, respectively. We merged the alleles from Populations A and B into a single group (Group AB) that contained 121 different alleles. In this case, the expected total number of different alleles, under the assumption that they were uniformly distributed at both locations, was 133. Afterwards, we compared the overlap of the alleles between Populations A and B, and then compared this with the overlap among the randomly generated groups (expA and expB). The expA and expB groups were generated by sampling with replacement and contained 184 and 188 elements, respectively, which were drawn from a pool that contained the calculated expected number of different alleles (133) (Table 1 and Materials and Methods).

**Table 1:** Number of *csd* alleles identified within the given honey bee groups and number of elements in randomly generated reference datasets.

	A	B	AB	redAB		
total number of sequenced alleles	184	188	372	273		
number of different alleles	85	74	121	121		
expected number of different alleles	111	78	133	152		
	**expA**	**expB**	**rA**	**rB**	**rAB**	**r-redAB**
number of picked elements (“alleles”)	184	188	184	188	372	273
number of different elements (“alleles”) in the pool	133	133	111	78	133	152

Upper part: the total number of identified sequences, different alleles and expected number of different alleles. The expected number of different alleles was calculated under the assumption of their uniform distribution within a given group. Lower part: number of elements constituting the reference random datasets and the number of different elements in the pool from which they were drawn.

**Figure 2:**
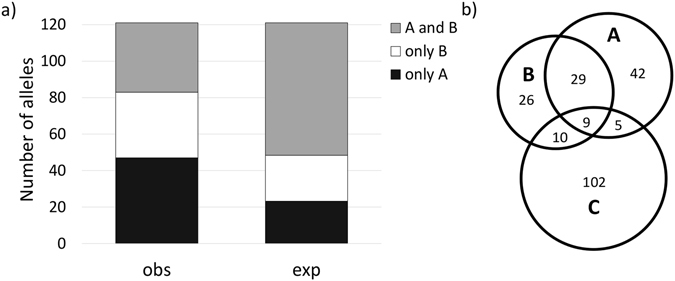
Overlap of *csd* alleles found in distinct geographical localisations. (**a**) Comparison of the overlap of *csd* alleles in the analysed groups (obs) with the overlap expected from the randomly generated groups of alleles (exp). The obs and exp distributions were compared using the chi-test and were found to be significantly different. (**b**) Venn diagram representing the overlap of *csd* alleles between the groups analysed in this study (A and B) and the set of 126 non-identical alleles described by Lechner *et al*. (C)^18^.

An analysis of the overlap among the alleles in Group AB showed that 47 alleles were present exclusively in Population A; 36 were present exclusively in Population B; and 38 were present in both populations (A and B). As shown in Fig. 2a, the observed distribution is significantly different (p = 1.4 × 10^−10^) from the distribution that was obtained from the analysis of expA and expB and reveals that the alleles common to both populations are underrepresented. This indicates that the spatial distribution of *csd* alleles is nonuniform.

To further substantiate this conclusion, we compared the alleles identified in Populations A and B with the alleles described in the paper by Lechner *et al*.^18^ (Population C). This dataset contained 154 *csd* sequences that represented 126 alleles of non-identical protein sequences. We found that the overlap between populations A, B and C was very low: we identified a total of 247 different alleles, only 9 of which were present in all three populations (Fig. 2b). Lechner’s *et al*. dataset encompassed *csd* alleles from honey bee populations located primarily in Kenya, but also from populations in other countries (USA, Brazil, Israel, France, Germany and Australia). As was described in the previous section, this dataset was used to determine the criteria for functional heterozygosity, which led to the estimation of the worldwide number of functionally distinct alleles. The number was estimated to be between 116 and 145. Regardless of our reservations concerning the validity of the concept of functionally distinct alleles, it is worth noting that the estimation methods used in both of the above analyses (Lechner’s and ours) were based on the assumption that the spatial distribution of *csd* alleles is uniform. Although the estimation of the number of alleles in our population (133) did not differ from the previous prediction (with the reservation that different criteria were adopted to identify distinct alleles), a comparison of the allele sequences between both datasets revealed a strikingly low degree of overlap. We also observed a significantly low overlap between the two local groups (A and B) than was expected under the assumption that the alleles were distributed uniformly, despite the fact that the analysed groups were separated by a relatively short distance. Moreover, taking into account the possibility that *csd* alleles could also be nonuniformly distributed within a given population (i.e. the frequencies of particular alleles may be unequal in a given local population) further complicates the matter of correct estimation of their worldwide number. The accuracy of such estimations would be particularly vulnerable to the existence of a large number of infrequent alleles^20^.

### Analysis of *csd* allele frequency

**Figure 3:**
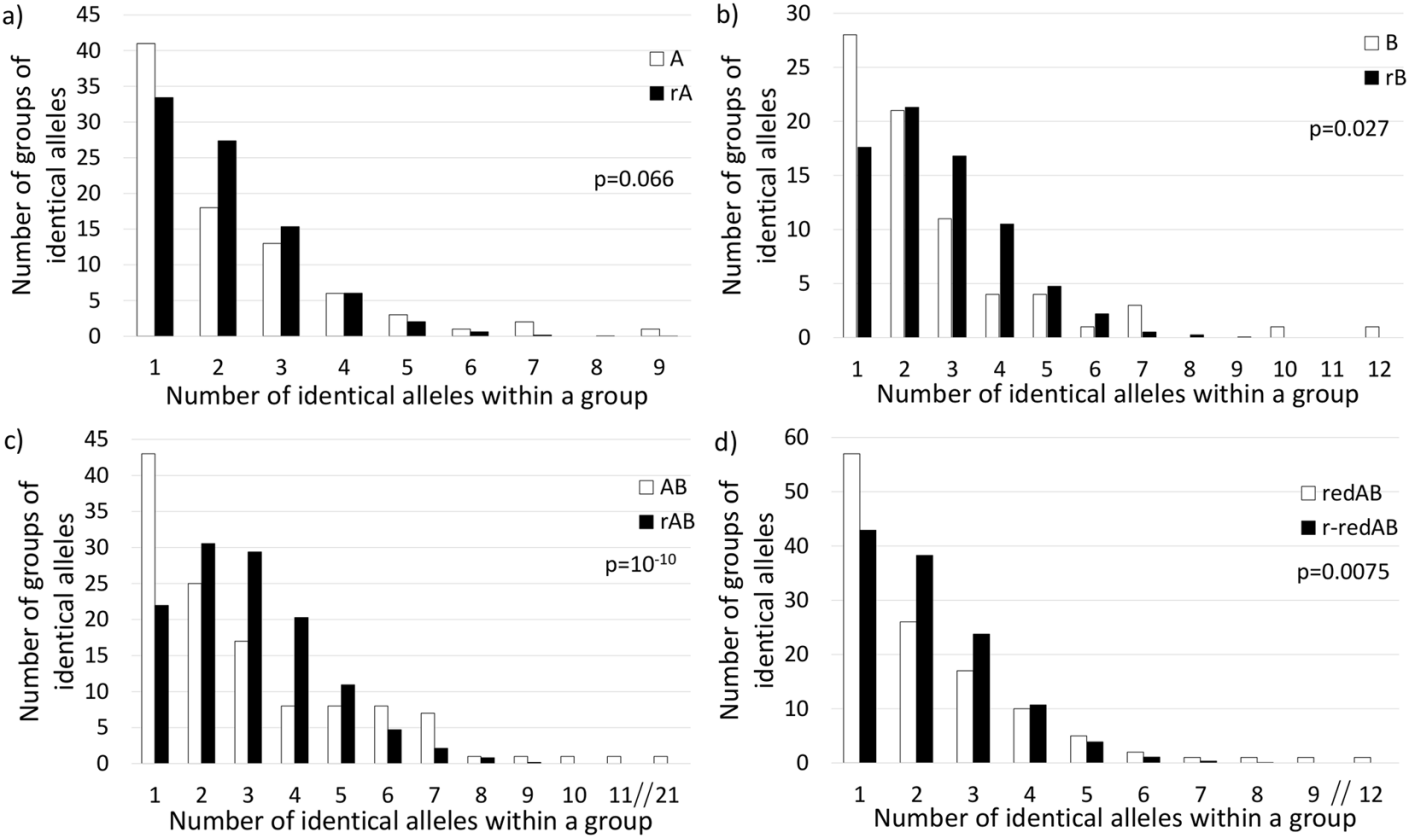
Frequency of *csd* alleles in the analysed populations. Histograms show the number of groups represented by a given number of identical alleles in Populations A (**a**), B (**b**) and AB (**c**) (white bars), which are compared to the distribution of alleles in the corresponding randomly generated datasets (rA, rB and rAB) (black bars). Histogram (**d**) shows the allele distribution in Population AB, which was calculated under the condition that any unique allele was counted only once if it was represented by several alleles identified in a single apiary (redAB), compared with the randomly generated dataset (r-redAB). The observed and random distributions were compared using the chi-test. The obtained *p* value is given in each diagram.

In order to determine whether the uneven distribution of *csd* alleles may have also resulted from a different frequency of the occurrence of particular alleles in a population, we calculated the number of groups that were represented by a given number of identical alleles (i.e. how many different alleles occurred in the analysed populations as singletons, doublets, triplets, etc.) in each population separately (A, B) and in Group AB. The obtained distributions were then compared to the distribution patterns calculated from the corresponding random sets (rA, rB and rAB, respectively) (Table 1, Fig. 3a–c). We noticed that the observed distributions differed substantially from the expected distributions (p = 0.066, p = 0.027 and p = 3 × 10^−10^, respectively) due to the higher incidence of alleles that occurred in the analysed populations only once (Fig. 3a–c) and ≥6 times (Fig. 3c). The higher incidence of the alleles that were identified several times during our analysis may have resulted from the fact that the bee colonies located in a single apiary can be genetically related (e.g. due to the swarms being located in the same apiary from which they emerged). To exclude this effect from our analysis, we recalculated our data under the condition that if a given unique allele was represented several times in single apiary, it was counted only once. Once the dataset was transformed in this manner, we then recalculated the expected diversity of *csd* alleles (152) in the Population AB (redAB) and generated the corresponding random sets of alleles (r-redAB). Afterwards, we analysed and compared the distribution of the allele frequency (Fig. 3d). The results indicated that, although the redAB and r-redAB datasets no longer differed in terms of the frequency of alleles that were represented several times, the redAB dataset was still highly enriched in singletons and differed significantly from its corresponding random dataset (p = 0.0075).

### Worldwide distribution of unique *csd* alleles in the analysed honey bee population

**Figure 4:**
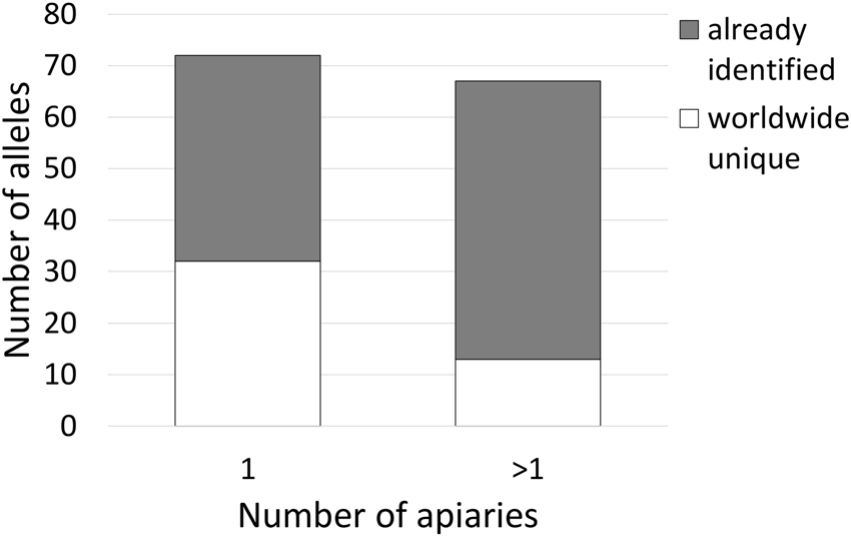
Identification of worlwide unique *csd* alleles and their distribution. Frequency of worldwide unique alleles (white bars) compared to the alleles that are already present in publicly available databases (grey bars) within the sets of *csd* alleles that were identified in this study and are present in 1 or >1 apiaries. These two distributions were compared using the chi-test and were found to be significantly different (p = 3 × 10^−6^).

In addition to the groups of apiaries that were analysed in the aforementioned time period, we also analysed one of the apiaries in Group A in 2013 and again in 2015. In this apiary, we identified the maternal and, to some extent, the paternal *csd* alleles. Together with this data, we analysed a total of 469 *csd* alleles and identified 139 different alleles, 72 of which were identified in one and 67 in more than one apiary. We used tBLASTn^21^ to compare our set of alleles with those available in the nucleotide collection at the National Center of Biotechnology Information (www.ncbi.nlm.nih.gov/nuccore) and found that 45 of these alleles had never been identified before. In accordance with the approach we used throughout our analysis, we identified unique worldwide as those that they were not identical in their amino acid sequences to any entry present in the database. Out of this set of worldwide unique alleles, 32 were identified in one and 13 in more than one apiary. This means that the probability of finding a worldwide unique *csd* allele in our population (0.44) was significantly greater (p = 9.4 × 10^−8^) in the subgroup that contains rare alleles (which were identified in one apiary) than in the subgroup that contains more frequent alleles (0.24) (Fig. 4). One possible explanation for such a distribution is that these infrequent worldwide unique alleles have been generated recently, and as such have not yet spread across the population.

In conclusion, we propose that *csd* alleles are distributed unevenly both in the terms of their spatial distribution and frequency within a given population. We base this proposal on our observations, which showed that the sets of *csd* alleles that were identified in various local populations overlapped to a strikingly lower degree than was expected under the assumption that the alleles are distributed uniformly. We have shown that the uneven distribution of the alleles stems from a large number of very infrequent alleles which presence is restricted to a single colony and/or apiary. Furthermore, we found that a large portion of these infrequent alleles had never been identified in the worldwide population. The occurrence of such alleles may indicate that they are detrimental for the analysed population at the particular stage of its of development. However, we find it more likely that these alleles were generated recently, and thus did not have enough time to spread across the population.

Taking these findings into account, the previous estimation of the total number of *csd* alleles should be revised. In our opinion, such an estimation is impossible to conduct due to the uneven distribution of the alleles and the large number of infrequent alleles^20^. The rapid generation of new alleles implies that the analysed local populations are in a state of dynamic equilibrium. This, in turn, along with the highly uncertain number of the alleles, makes it impossible to reliably determine values such as the rate at which new alleles are being generated or the effective size of the population using the methods that were described in previous studies^13, 18^. It is also worth noting at this point that in these studies, the values of these parameters have differed very significantly, depending on the assumed total number of alleles. Therefore, it seems that the rate at which new *csd* alleles are generated should be determined through direct observations. This should be possible considering the postulated similarity between the HVR and the microsatellite regions, and the very high estimated rate of mutations that are caused by a DNA slippage (10^−2^–10^−5^)^22, 23^.

Taking into account the significance of the extremely high frequency of the generation of new *csd* alleles that has been postulated, we speculate that in addition to its role in sex determination, *csd* may play a role in controlling the balance between inbreeding and outbreeding in honey bee reproduction^11, 24, 25^. Given that the social organisation of a honey bee colony allows for the reproduction of only a very limited number of individuals relative to the total number of individuals that can be sustained in a given environment, the effective size of the population is small^5, 26^. Such conditions imply that the degree of inbreeding within a local population is high. Consequently, a high rate of *csd* allele generation would be required in order to protect such a population against the overgeneration of diploid males. On the other hand, an excessive rate of new *csd* allele generation could lead to the generation of organisms that are heterozygous for *csd*, but otherwise have very similar haplotypes. This would increase the chances of the emergence of a set that is homozygous for genes other than *csd*, which in turn could be detrimental for a given population if deleterious recessive mutations were to occur. Thus, if the rate of the generation of *csd* alleles is indeed high, then *csd* could be used as a prompt to restrict immediate incest and the reduction of genetic diversity; but, at the same time, would allow for a relatively high degree of inbreeding. The effect of the optimal inbreeding/outbreeding equilibrium can be observed even in human populations, where the reproductive potential does not grow with the genetic distance between couples and is the highest between second and third cousins^27^.

Further research, in particular an accurate determination of the frequency and mechanisms that underlie the generation and spreading of the *csd* alleles in the honey bee population, is needed in order to investigate the role of *csd* diversity in the process of honey bee reproduction in more detail.

## Materials and Methods

### Sample collection

Drone pupae and larvae were collected late May and early June 2014 from 6 apiaries localized ~20 km from Wroclaw, Poland. The apiaries were located in a area of 3.5 km radius. In the next season (May and June 2015) samples were collected from 12 apiaries localized near Ladek-Zdroj, Poland, scattered in a area of approx. 20 km radius and 100 km apart from the first group of apiaries. The analyzed colonies were kept in apiaries with little to none management that could affect the genetic diversity, except for swarming prevention by removing the queen larvae. The collected samples were stored in 95% ethanol at −20 °C.

### DNA isolation and amplification

Genomic DNA was isolated using binding to silicon dioxide in the presence of chaotropic agents. A fragment of the tissue was incubated for 2 hours at 56 °C in 200 ul of buffer (100 mM Tris-HCl pH 8.0, 5 mM EDTA, 0.3% SDS, 200 mM NaCl) containing 15 ul of proteinase K (10 mg/ml). 12 ul of 3 M sodium acetate was added and the crude lysate was incubated for 15 minutes on ice. After the extraction with equal volume of chloroform, 250 ul of 6 M sodium iodide and 16 ul of 10% silicon dioxide (Sigma-Aldrich S5631) suspension were added to the collected water phase which was then incubated with occasional mixing for 5 minutes at room temperature. After centrifugation, the pellet was washed with 300 ul of washing solution (50% ethanol, 10 mM Tris-HCl pH 7.5, 100 mM NaCl and 1 mM EDTA), resuspended in 25 ul of water and incubated at 56 °C for 5 minutes. After centrifugation, 2 ul of the supernatant was used in a PCR reaction (30 cycles, 51 °C annealing) using *csd* specific primers^28^ (csdF1: 5′AGACrATATGAAAAATTACACAATGA, csdR1: 5′TCATwTTTCATTATTCA). The amplification products were visualized in gel electrophoresis and punched as agarose slabs which were then incubated with 30 ul of water at 56 °C for 10 minutes. 1 ul of the solution was used in nested-PCR reaction (25 cycles, 51 °C annealing) using labelled (Hex and 6-FAM for csdF2 and csdR2, respectively, for further use in RFLP) or unlabelled (for sequencing) primers^28^ encompassing ~300 bp fragment containing the hypervariable region of *csd* gene (csdF2: 5′-TATCGAGAAAsATCGAAAGAACGAT, csdR2: 5′-ATTGAAATCCAAGGTCCCATTGGT). As inferred from the subsequent sequencing, the amplification products were homogenous and the primer sets used did not amplify *fem* gene.

### Restriction fragment length polymorphism (RFLP) analysis

3 ul of the second amplification product was digested in 10 ul of reaction mixture containing 5 U of VspI restriction enzyme (Thermo Scientific) and incubated for 1 hour at 37 °C. 1 ul of the digestion product was denatured in the presence of Hi-Di formamide and 0.5 ul of 350 bp Rox standard (Life Technologies) and subjected to capillary electrophoresis on ABI Prism 310 apparattus. The size of the restriction fragments were assessed using GeneMarker software.

### Sanger sequencing

Prior to the sequencing reaction the products (15 ul) of the second amplification were digested with exonuclease I (0.5 U, Thermo Scientific) and Shrimp Alkaline Phosphatase (0.25 U, New England Biolabs) for 30 minutes at 37 °C. The enzymes were inactivated by denaturation (5 minutes, 95 °C). 3 ul of the DNA was used in cycle sequencing reaction (10 ul) containing: 1.9 ul 5× sequencing buffer, 0.5 ul BigDye Terminator Cycle Sequencing mix and 0.65 ul 5 uM csdF2 primer. The sequencing products were precipitated with 75% isopropanol in the presence of 1 ul of glycogen (10 mg/ml), resuspended in 15 ul of Hi-Di formamide, denatured and subjected to capillary electrophoresis (ABI Prism 310). Data was analyzed using ABI Sequence Analysis software (v3.3). Alleles that have been identified only once (singletons) were re-analyzed starting from the DNA isolation step. All non-redundant seqeunces have been resequenced using csdR2 primer.

### Data analysis

The nucleotide sequences of *csd* gene fragments encoding the HVR and part of the proline-rich region of csd protein (Fig. 1a) were *in silico* translated in ExPASy translate tool^29^. The pairwise alignments of all non identical sequences were performed in Jalview^30^ using the default alignment parameters, however artificial extensions to the sequences (7 amino acids on both ends of each sequence) has been added to prevent introducing gaps on the sequence ends which are known to include conserved amino acid positions. The resulting data was analysed in Microsoft Excel to count the differences in HVR length and number of amino acid substitutions.

The expected minimal number of different *csd* alleles in all analyzed datasets was calculated using a mathematical model which is based on the theory of Markov processes. This assumption follows immediately from observations that if we assume that the total number of different alleles is equal to *N* and the probability of finding any alleles is $\frac{1}{N}$ then stochastic process described by {*Y*_*i*_} where *Y*_*n*_ is the number of different alleles collected after *n* units of time will be a Markov chain. This Markov chain is described on the state space *S* = {0, 1, 2, 3, …, *N*} with transition probability matrix:

$${P}_{N}={\{{p}_{N}(i,j)\}}_{1\le i,j\le N}=[\begin{array}{ccccccc}0 & 1 & 0 & \cdots  & \cdots  & \cdots  & 0\\ 0 & \frac{1}{N} & \frac{N-1}{N} & 0 & \cdots  & \cdots  & 0\\ 0 & 0 & \frac{2}{N} & \frac{N-2}{N} & 0 & \cdots  & 0\\ \vdots  & \vdots  & \ddots  & \ddots  & \ddots  & \ddots  & \vdots \\ 0 & \cdots  & \cdots  & \cdots  & 0 & \frac{N-1}{N} & \frac{1}{N}\end{array}].$$

However, *N* is unknown and needs to be estimated. Therefore, we decided to use a specific estimation method. This procedure is based on observation that for fixed *N*, *U* - number of drawing unique alleles and *D* - the total number of draws we are able to compute ${Q}_{N}^{D}(U)=1-{\sum }_{i=1}^{U}{p}_{N}^{D}(1,i)$. It is clear that ${Q}_{N}^{D}(U)$ is the probability that more than *U* - unique alleles are observed in the case when *N* is the total number of different *csd* alleles, *D* is a number of drawing. It seems reasonable to assume that ${Q}_{N}^{D}(U)$ should not take a high value. In our investigation we fixed ${Q}_{N}^{D}(U)=0,05$ and assume that estimated number of different *csd* alleles is equal to $\mathop{{\rm{\arg }}\,{\rm{\min }}}\limits_{N}({Q}_{N}^{D}(U)\le 0,05)$.

The reference datasets used in the analysis of the distribution and frequency of the alleles in the given groups and subgroups were generated by sampling with replacement using Microsoft Excel. Specifically, a given number of elements (corresponding to the number of alleles present in the analysed group) were drawn from a group containing *n* different elements (*n* = number of different alleles estimated to be present in the analysed group, see above). The number of each specific element in the generated set was counted and then the number of groups represented by a given number of identical elements was determined. The mean values obtained from 100 sets were used as expected values and compared with the observed values using chi-test (Microsoft Excel).

## References

[1] Dzierzon, J. Gutachten über die von Herrn Direktor Stöhr im ersten und zweiten Kapitel des General-Gutachtens aufgestellten Fragen. *Eichstädter Bienenzeitung* **1**, 109–113, 119–121 (1845).

[2] van Wilgenburg E, Driessen G, Beukeboom LW (2006). Single locus complementary sex determination in Hymenoptera: an “unintelligent” design?. Frontiers in Zoology.

[3] Cole BJ (1983). Multiple Mating and the Evolution of Social Behavior in the Hymenoptera. Behav Ecol Sociobiol.

[4] Zayed A, Packer L (2005). Complementary sex determination substantially increases extinction proneness of haplodiploid populations. Proc. Natl. Acad. Sci..

[5] Graur D (1985). Gene Diversity in Hymenoptera. Soc. Study Evol..

[6] Beye M, Hasselmann M, Fondrk MK, Page RE, Omholt SW (2003). The gene csd is the primary signal for sexual development in the honeybee and encodes an SR-type protein. Cell.

[7] Mackensen O (1951). Viability and sex determination in the honeybee (*Apis mellifera* L.). Genetics.

[8] Woyke, J., Knytel, A. The chromosome number as proof that drones can arise from fertilized eggs of the honeybee. *J*. *Apic*. *Res*. **5**, 149–154 (1966).

[9] Woyke, J. What happens to diploid drone larvae in a honeybee colony. *J*. *Apic*. *Res*. **2**, 73–75 (1963).

[10] Zayed A, Packer L (2007). The population genetics of a solitary oligolectic sweat bee, Lasioglossum (Sphecodogastra) oenotherae (Hymenoptera: Halictidae). Heredity (Edinb)..

[11] Page REJ, Marks RW (1982). The population genetics of sex determination in honey bees: random mating in closed populations. Heredity (Edinb)..

[12] Cho S, Huang ZY, Green DR, Smith DR, Zhang J (2006). Evolution of the complementary sex-determination gene of honey bees: Balancing selection and trans-species polymorphisms. Genome Res..

[13] Hasselmann M (2008). Evidence for Convergent Nucleotide Evolution and High Allelic Turnover Rates at the *complementary sex determiner* Gene of Western and Asian Honeybees. Mol. Biol. Evol..

[14] Gempe T (2009). Sex determination in honeybees: two separate mechanisms induce and maintain the female pathway. PloS Biol..

[15] Nissen I, Müller M, Beye M (2012). The Am-tra2 gene is an essential regulator of female splice regulation at two levels of the sex determination hierarchy of the honeybee. Genetics.

[16] Hasselmann M, Lechner S, Schulte C, Beye M (2010). Origin of a function by tandem gene duplication limits the evolutionary capability of its sister copy. Proc. Natl. Acad. Sci. USA.

[17] Biewer, M., Schlesinger, F. & Hasselmann, M. The evolutionary dynamics of major regulators for sexual development among Hymenoptera species. *Front*. *Genet*. **6**(124) (2015). 10.3389/fgene.2015.00124 PMC4392698 25914717

[18] Lechner S (2013). Nucleotide Variability at Its Limit? Insights into the Number and Evolutionary Dynamics of the Sex-Determining Specificities of the Honey Bee Apis mellifera. Mol. Biol. Evol..

[19] Beye M (2013). Gradual molecular evolution of a sex determination switch through incomplete penetrance of femaleness. Curr Biol..

[20] Bunge J, Fitzpatrick M (1993). Estimating the Number of Species: A Review. Journal of the American Statistical Association.

[21] Altschul SF, Gish W, Miller W, Myers EW, Lipman DJ (1990). Basic local alignment search tool. J. Mol. Biol..

[22] Schlotterer C, Tautz. D (1992). Slippage synthesis of simple sequence DNA. Nucleic Acid Res..

[23] Ellegren H (2004). Microsatellites: simple sequences with complex evolution. Nat Rev Genet..

[24] Yokoyama S, Nei M (1979). Population dynamics of sex-determining alleles in honey bees and self-incompatibility alleles in plants. Genetics.

[25] Cook JM, Crozier RH (1995). Sex determination and population biology in the hymenoptera. Trends Ecol. Evol..

[26] Frankham R (1995). Effective population size/adult population size ratios in wildlife: a review. Genet. Res..

[27] Helgason, A., Palsson, S., Guobjartsson D. F., Kristjansson, T., Stefansson, K. An Association Between the Kinship and Fertility of Human Couples. *Science* **319**, 813–816 (2008). 10.1126/science.1150232 18258915

[28] Hyink O, Laas F, Dearden PK (2012). Genetic tests for alleles of complementary-sex-determiner to support honeybee breeding programmes. Apidologie.

[29] Gasteiger E (2003). ExPASy: The proteomics server for in-depth protein knowledge and analysis. Nucleic Acids Res..

[30] Waterhouse AM, Procter JB, Martin DMA, Clamp M, Barton GJ (2009). Jalview Version 2-A multiple sequence alignment editor and analysis workbench. Bioinformatics.

